# Supplementary material for: Angelica sinensis polysaccharide as potential protectants against recurrent spontaneous abortion: focus on autophagy regulation
Source: Front Med (Lausanne). 2025 Jan 15;12:1522503. doi: 10.3389/fmed.2025.1522503 (PMC11774876; doi:10.3389/fmed.2025.1522503)
Supplement: Supplementary material S1 — The main instruments used during the LC-MS process, along with their models/specifications and manufacturers. [file Supplementary_file_1.zip › Metabolomics sequencing data FC1.2/8.代谢通路分析/Exposure_Ctrl/all/气泡图-top20.pdf]

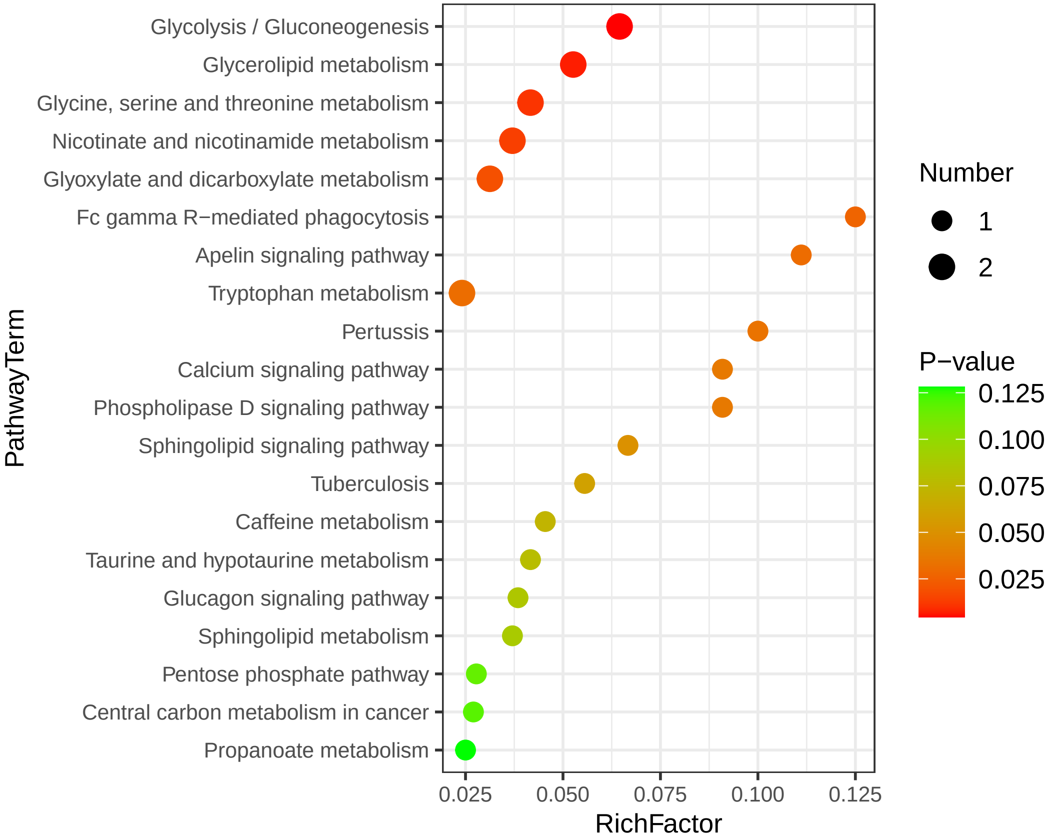
Glycolysis / Gluconeogenesis
Glycerolipid metabolism
Glycine, serine and threonine metabolism
Nicotinate and nicotinamide metabolism
Glyoxylate and dicarboxylate metabolism
Fc gamma R−mediated phagocytosis
Apelin signaling pathway
Tryptophan metabolism
Pertussis
Calcium signaling pathway
Phospholipase D signaling pathway
Sphingolipid signaling pathway
Tuberculosis
Caffeine metabolism
Taurine and hypotaurine metabolism
Glucagon signaling pathway
Sphingolipid metabolism
Pentose phosphate pathway
Central carbon metabolism in cancer
Propanoate metabolism
PathwayTerm
0.025
0.050
0.075
0.100
0.125
RichFactor
Number
1
2
P−value
0.125
0.100
0.075
0.050
0.025
